# Patterns and trends in sources of information about sex among young people in Britain: evidence from three National Surveys of Sexual Attitudes and Lifestyles

**DOI:** 10.1136/bmjopen-2015-007834

**Published:** 2015-03-04

**Authors:** Clare Tanton, Kyle G Jones, Wendy Macdowall, Soazig Clifton, Kirstin R Mitchell, Jessica Datta, Ruth Lewis, Nigel Field, Pam Sonnenberg, Amy Stevens, Kaye Wellings, Anne M Johnson, Catherine H Mercer

**Affiliations:** 1 Research Department of Infection and Population Health, University College London, London, UK; 2 Centre for Sexual and Reproductive Health Research, London School of Hygiene and Tropical Medicine, London, UK; 3 NatCen Social Research, London, UK

**Keywords:** EDUCATION & TRAINING (see Medical Education & Training), SEXUAL MEDICINE, PUBLIC HEALTH

## Abstract

**Objective:**

To assess progress in meeting young people's sex education needs in Britain by examining the current situation and changes over the past 20 years in sources of information about sexual matters and unmet information needs.

**Design:**

Cross-sectional probability sample surveys.

**Setting:**

British general population.

**Participants:**

3869 men and women aged 16–24 years, interviewed 2010–2012 for the third National Survey of Sexual Attitudes & Lifestyles (Natsal-3), compared with 16–24 year-olds in Natsal-1 (1990–1991; 792 men and women) and Natsal-2 (1999–2001; 2673 men and women).

**Main outcome measures:**

Reported source of information about sexual matters, unmet information needs and preferred source of additional information.

**Results:**

Between 1990 and 2012, the proportion citing school lessons as their main source of information about sexual matters increased from 28.2% (95% CI 24.6 to 32.1) to 40.3% (95% CI 38.6 to 42.1). In 2010–2012, parents were reported as a main source by only 7.1% (95% CI 5.8 to 8.7) of men and 14.1% (95% CI 12.6 to 15.7) of women and, for women, were less commonly reported than in 1999–2001 (21.7%; 95% CI 19.6 to 24.0). Most young people reported not knowing enough when they first felt ready for sexual experience (68.1% men, 70.6% women), and this did not change substantially over time. They wanted more information about psychosexual matters (41.6% men, 46.8% women), as well as sexually transmitted infections (27.8% men, 29.8% women) and, for women, contraception (27.5%). Young people primarily wanted this information from school, parents or health professionals.

**Conclusions:**

Over the past 20 years, young people have increasingly identified school lessons as their main source of information about sex, although they continue to report needing more information on a broad range of topics. The findings support the expressed need for improved sex and relationships education in schools alongside greater involvement of parents and health professionals.

**Strengths and limitations of this study**

- This study uses data from serially-conducted British population-based surveys with nationally representative samples to explore how young people learn about sex and how this has changed over time.
- Using data from the three surveys to examine change over time (as opposed to looking at change across the life course in one survey) is likely to minimise recall and recasting biases.
- After weighting, our sample was comparable with the 2011 census on key demographics, however, we cannot be sure that those who participated are representative of the population.
- Our study demonstrates that over the past 20 years there have been substantial increases in the proportion of young people citing school as their primary source of information about sex.

## Introduction

The role of schools in providing information about sexual matters is much debated, with some arguing that school-based sex and relationships education (SRE) may accelerate the onset of sexual activity and that parents should provide the information (giving them control over what is delivered and when).^1^ The biological elements of sex education (biological aspects of puberty, reproduction and sexually transmitted infections, including HIV/AIDS) must be taught to all pupils through National Curriculum Science, which is compulsory in state-maintained schools in England (although not in academies and free schools). State-maintained secondary schools must now, in addition, teach the wider personal and social aspects of sex and relationships and any state-funded school that provides SRE must have ‘due regard’ to the government guidance.^2–4^ However, there is no statutory curriculum so schools are encouraged to develop their own programmes of study. Personal, social, health and economic education (PSHE), which includes the broader aspects of relationships education, is not statutory. Relationships education has been compulsory since 2003 in Wales;^5^ ^6^ there is no statutory requirement for schools in Scotland to teach SRE but guidance exists.^7^

There is evidence that receipt of SRE impacts positively on sexual behaviour and is associated with positive sexual health outcomes.^8–10^ Yet the quality of provision and coverage of topics is variable in Britain.^11–15^ In 2013, the Office for Standards in Education, Children's Services and Skills (Ofsted) were of the view that SRE required improvement in over a third of English schools and in almost half of English secondary schools.^16^ However, young people learn about sex in a variety of ways^17–19^ and the landscape in which they are learning is changing. The range and relative importance of sources have changed over time. Most notably, in recent years there has been a rapid evolution in technology and internet access, which creates both opportunities and challenges for young people's sexual well-being. Although it is now easy to access high-quality sexual health information online, young people are also increasingly able to access sexually-explicit material and there is concern that this may create unrealistic expectations about sex and relationships.^13^ ^20^ Young people increasingly conduct much of their social lives online which raises concerns about online safety.^21^ Advice to supplement the government's SRE Guidance^2^ attempts to address this,^22^ recommending that teaching should also include the impact of pornography, safe use of technology, sexual consent, exploitation and abuse and violence in relationships.

Up-to-date evidence is needed about the range and relative importance of different information sources for young people, how well these sources meet their information needs, as well as their preferred sources and knowledge deficits, to ensure that young people are equipped with the knowledge they need to experience a happy and healthy sex life. This paper seeks to address this evidence gap using data reported by young people (16–24 years), in the third British National Survey of Sexual Attitudes and Lifestyles (Natsal-3), a probability sample survey conducted in 2010–2012, as well as data from the previous two decennial Natsal surveys to examine how learning about sex has changed over the past 20 years.

## Methods

### Participants and procedure

The Natsal probability sample surveys to date have been carried out approximately decennially in 1990–1991 (Natsal-1),^23^ ^24^ 1999–2001 (Natsal-2)^25^ ^26^ and 2010–2012 (Natsal-3).^27^ In all three surveys, households were selected using stratified probability sampling, from which one eligible individual, resident in Britain, was selected at random and invited to participate.

Natsal-3 interviewed 15 162 men and women aged 16–74 years (1729 men and 2140 women aged 16–24 years). The overall response rate was 57.7%. Full details of the methodology have been published previously.^27^ ^28^ Participants were interviewed using computer-assisted personal interviewing (CAPI) with computer-assisted self-interview for the more sensitive questions.

### Measures

Questions on learning about sex were asked face-to-face in the CAPI section of the questionnaire, of all participants. Participants were asked ‘When you were growing up, in which of the ways listed on this card did you learn about sexual matters?’ and, subsequently, ‘From which did you learn most?’. For the first question multiple sources could be given but for the second question, one main source. Participants were then asked ‘Looking back to the time when you first felt ready to have some sexual experience yourself, is there anything on this list that you now feel you ought to have known more about?’. Those who had unmet information needs were asked ‘How, or from whom, would you have liked to learn more about those sexual matters, please choose just one or two from this list?’. For all of these questions participants were given showcards listing the answer options (box 1).

Box 1: **Question answer options**

When you were growing up, in which of the ways listed on this card did you learn about sexual matters?*

 Mother (including step or adoptive)          Television/radio/DVDs/videos

 Father (including step or adoptive)          Books/magazines/newspapers

 Brother(s)/sister(s) (including half, step or adoptive)    **Internet—sexual advice websites**

 Lessons at school                **Internet—pornographic websites**

 Friends of about my own age   **         Internet—other**

 First boyfriend/girlfriend or sexual partner       **Pornographic magazines/films**

 Doctor, nurse or clinic              Other (please say how)

Looking back to the time you first felt ready to have some sexual experience yourself, is there anything on this list that you now feel you ought to have known more about?

 How girls' bodies develop   How to make sex more satisfying

 How boys' bodies develop   How to be able to say ‘no’

 How a baby is born      Sexual feelings, emotions and relationships

 Sexual intercourse      Sexually transmitted infections (eg, VD/chlamydia/HIV)

 **Other sexual practices**   ** Safer sex**

 Contraception, birth control   **How to use a condom correctly**

 Homosexuality, lesbianism   All of them

 Masturbation        None—not ready for sexual experience

*Same answer options for questions on main and preferred source of information.

Bold text indicates answer options which were new for Natsal-3.

Similar measures and procedures were used in Natsal-1 and Natsal-2. While response options were largely consistent across all three surveys, sources of information about sex were updated to be relevant in the current social context, particularly bearing in mind the availability of the internet, and the list of topics on which additional information may have been needed were updated (box 1).

### Statistical analysis

Complex survey analysis was carried out in Stata (V.13.0) accounting for stratification, clustering and weighting of the data. Analysis of Natsal-3 data is restricted to participants aged 16–24 in order to provide a contemporary picture of how young people learn about sex. Specifically, we describe any, and main, reported sources of information about sexual matters, unmet information needs and the preferred source of this information, by gender. In addition, as young people's access to pornography is currently of great interest,^13^ ^20^ we compare men who reported pornography as one of their sources of information about sex with those who did not, both in terms of their other and main sources, and their unmet information needs using univariate logistic regression. The number of women reporting pornography as an information source (n=53) is too small to permit detailed exploration.

Finally, we use data from 16–24-year-olds in all 3 Natsal surveys (362 men and 430 women were asked these questions in Natsal-1; 1231 men and 1442 women in Natsal-2) to describe changes in the reporting of main sources of information about sexual matters and unmet information needs.

All p values for associations, with gender or survey number, were assessed using univariate logistic regressions.

## Results

### All sources of information about sexual matters reported in Natsal-3

Table 1 shows the proportion of young people in Natsal-3 who reported different sources of information about sex, and, of these, their main source of information. School was most commonly reported as a source of information about sexual matters by around 80% of participants in Natsal-3 (table 1), followed by friends (of about their own age), reported by two-thirds of the sample. Except for friends, siblings and media, there were gender differences in the sources reported: women were more likely than men to cite a parent as a source (43.4% vs 27.2% of men; p<0.0001), most commonly their mother (42.7%), while among men, a similar proportion cited either their mother (19.8%) or their father (17.6%). Women were more likely than men to report health professionals (doctor/nurse/clinic), although few reported this source, and men were more likely than women to cite the internet (excluding pornography), their first sexual partner (first girlfriend/boyfriend/sexual partner) and pornography. In total, 23.9% of men compared with only 2.2% of women, cited pornography as a source (19.4% from internet-based pornography and 9.2% from pornographic magazines/films).

**Table 1:** Any and main sources of information about sex in individuals aged 16–24 years in Natsal-3, by gender

	Men		Women		p Value for association with gender
	Per cent	95% CI	Per cent	95% CI	
Any source					
Lessons at school	78.5	(76.1 to 80.6)	81.9	(79.9 to 83.7)	0.0216
Friends of about own age	65.9	(63.3 to 68.5)	66.2	(63.8 to 68.5)	0.8822
First girlfriend/boyfriend/sexual partner	28.0	(25.6 to 30.5)	20.7	(18.8 to 22.6)	<0.0001
Parents	27.2	(24.9 to 29.6)	43.4	(41.0 to 45.8)	<0.0001
Mother	19.8	(17.8 to 22.0)	42.7	(40.3 to 45.2)	<0.0001
Father	17.6	(15.7 to 19.7)	6.9	(5.8 to 8.2)	<0.0001
Brother/sister	10.7	(9.0 to 12.7)	11.3	(9.9 to 12.8)	0.6336
Doctor/nurse/clinic	6.9	(5.7 to 8.4)	15.2	(13.6 to 17.0)	<0.0001
Media	47.0	(44.4 to 49.7)	49.0	(46.7 to 51.4)	0.2426
Books/magazines/newspapers	20.1	(18.1 to 22.4)	29.0	(26.8 to 31.3)	<0.0001
TV/radio/DVDs/videos	39.5	(36.9 to 42.1)	35.4	(33.3 to 37.6)	0.0136
Internet (excluding pornography)	28.6	(26.2 to 31.2)	14.0	(12.4 to 15.7)	<0.0001
Internet-sexual advice websites	19.1	(17.1 to 21.3)	9.5	(8.2 to 11.0)	<0.0001
Internet-other	14.1	(12.3 to 16.0)	6.3	(5.2 to 7.6)	<0.0001
Any pornography	23.9	(21.6 to 26.4)	2.2	(1.7 to 3.0)	<0.0001
Internet-pornographic websites	19.4	(17.3 to 21.8)	1.8	(1.3 to 2.5)	<0.0001
Pornographic magazines/films	9.2	(7.8 to 10.9)	0.9	(0.5 to 1.4)	<0.0001
Other	1.5	(1.0 to 2.2)	1.7	(1.1 to 2.4)	0.7092
Main sources					
Lessons at school	39.4	(36.8 to 42.0)	41.3	(39.0 to 43.7)	0.2708
Friends of about own age	24.1	(21.9 to 26.5)	24.1	(22.1 to 26.2)	0.9944
First girlfriend/boyfriend/sexual partner	11.5	(9.8 to 13.3)	5.4	(4.4 to 6.6)	<0.0001
Mother	4.3	(3.3 to 5.5)	13.5	(12.0 to 15.2)	<0.0001
Father	2.8	(2.0 to 4.0)	0.5	(0.3 to 0.9)	<0.0001
Brother/sister	2.1	(1.5 to 3.0)	2.2	(1.6 to 2.9)	0.8993
Doctor/nurse/clinic	0.9	(0.5 to 1.6)	2.6	(1.9 to 3.6)	0.0010
Media	6.6	(5.3 to 8.0)	7.6	(6.4 to 9.0)	0.3090
Internet (excluding pornography)	4.1	(3.2 to 5.3)	1.9	(1.3 to 2.8)	0.0009
Pornography	3.4	(2.6 to 4.5)	0.2	(0.1 to 0.5)	<0.0001
Other	0.9	(0.5 to 1.6)	0.8	(0.4 to 1.3)	0.7310
Unweighted, weighted denominators	1729, 1238		2140, 1207		

### Main source of information about sexual matters

School was also the most common main source for both men (39.4% (36.8 to 42.0)) and women (41.3% (39.0 to 43.7); table 1), followed by friends (24.1%). Again, women were more likely than men to report a parent as their main source, usually their mother (13.5% (12.0 to 15.2)). Only 4.3% (3.3 to 5.5) of men reported their mother, and fewer still (2.8% (2.0 to 4.0)) their father. In total 11.5% (9.8 to 13.3) of men reported their first sexual partner as their main source, a higher proportion than among women (5.4% (4.4 to 6.6)). Men were more likely than women to cite the internet (excluding pornography) as their main source (4.1% (3.2 to 5.3) vs 1.9% (1.3 to 2.8)). A small minority of men (3.4% (2.6 to 4.5)) and very few women (0.2% (0.1 to 0.5)) reported pornography as their main source.

### Unmet information needs

Most men (68.1% (65.4 to 70.7)) and women (70.6% (68.1 to 73.0)) felt that that they ought to have known more when they first felt ready to have some sexual experience (table 2). Almost half the respondents felt they ought to have known more about topics related to reducing health risks of sexual behaviour (46.0% of men and 49.1% of women; table 2) or psychosexual issues (41.6% of men and 46.8% of women). Among risk reduction topics, ‘sexually transmitted infections’ (27.8% of men and 29.8% of women) was most commonly reported. Among psychosexual topics, respondents most commonly cited ‘sexual feelings, emotions, relationships’ (23.2% of men and 29.7% of women). Although a deficiency of biological information was less commonly mentioned, just over one in five men and women felt they ought to have known more about this aspect of sexual well-being.

**Table 2:** Topics individuals aged 16–24 years in Natsal-3 felt they ought to have known more about when they first felt ready to have some sexual experience, by gender

	Men		Women		p Value for association with gender
	Per cent	95% CI	Per cent	95% CI	
**Any topic**	**68.1**	**(65.4–70.6)**	**70.6**	**(68.1–73.0)**	**0.1467**
**Any biological topic**	**22.1**	**(19.9–24.6)**	**22.6**	**(20.5–24.7)**	**0.8030**
How girls’ bodies develop	11.6	(9.8–13.6)	10.7	(9.2–12.3)	0.4745
How boys’ bodies develop	8.2	(6.8–10.0)	7.5	(6.3–9.0)	0.5165
How a baby is born	7.5	(6.1–9.2)	8.5	(7.2–9.9)	0.3726
Sexual intercourse	12.6	(10.8–14.5)	13.8	(12.2–15.6)	0.3380
**Any risk-reduction topic**	**46.0**	**(43.2–48.9)**	**49.1**	**(46.5–51.7)**	**0.1119**
Safer sex	19.1	(16.9–21.5)	17.4	(15.6–19.4)	0.2676
Contraception/birth control	19.3	(17.2–21.6)	27.5	(25.3–29.8)	<0.0001
Correct condom use	14.4	(12.5–16.5)	15.1	(13.4–17.0)	0.5961
Sexually transmitted infections	27.8	(25.3–30.4)	29.8	(27.6–32.2)	0.2341
**Any psychosexual topic**	**41.6**	**(38.8–44.3)**	**46.8**	**(44.2–49.4)**	**0.0055**
How to make sex more satisfying	19.7	(17.4–22.1)	15.4	(13.6–17.3)	0.0054
How to be able to say ‘no’	10.7	(9.0–12.8)	16.6	(14.8–18.5)	<0.0001
Sexual feelings, emotions, relationships	23.2	(21.0–25.6)	29.7	(27.3–32.1)	0.0002
Homosexuality/lesbianism	8.5	(7.0–10.2)	8.5	(7.2–10.1)	0.9444
Other sexual practices	9.0	(7.5–10.8)	9.7	(8.4–11.2)	0.5599
Masturbation	6.6	(5.3–8.2)	8.3	(7.0–9.8)	0.1031
Unweighted, weighted denominators*	1546, 1115		1909, 1070		

*176 (119 weighted) men and 222 (131 weighted) women did not feel ready for a sexual experience and did not answer this question. Further denominator changes are due to missing data. Bold typeface indicates grouped variables.

Overall, 8.5% of both men and women wanted more information on same-sex relationships. This was higher in those who self-identified as gay/lesbian, bisexual or other: 39.7% (26.5 to 54.5) of 60 men, and 28% (18.0 to 40.8) of 94 women. Women self-identifying as gay/lesbian, bisexual or other were more likely to report they ought to have known more (85.4% (76.4 to 91.3) vs 69.8% (67.2 to 72.2) of women who identified as heterosexual). There was no substantial difference for men (72.6% (58.4 to 83.4) vs 67.9% (65.2 to 70.5), respectively), although numbers in the former group were small.

Information needs varied little by gender. Women were more likely to report having wanted to know more about ‘contraception/birth control’ (27.5% (25.3 to 29.8) vs 19.3% (17.2 to 21.6)) and ‘how to say no’ (16.6% (14.8 to 18.5) vs 10.7% (9.0 to 12.8)) than men. Men were more likely to report feeling they ought to have known more about ‘how to make sex more satisfying’ (19.7% (17.4 to 22.1) vs 15.4% (13.6 to 17.3)).

The proportion of young people who would have liked further information was lowest among those reporting school as their main source (62.0% for men and 62.7% for women; table 3).

**Table 3:** Percentage of individuals aged 16–24 years in Natsal-3 reporting not knowing enough when they first felt ready to have some sexual experience, by main source of information and gender

	Men			Women			p Value for association with gender
	Per cent	95% CI	Denominators*	Per cent	95% CI	Denominators*	
All	68.1	(65.4 to 70.7)	1729, 1238	70.6	(68.1 to 72.9)	2140, 1207	0.1467
Main source of sex education							
Lessons at school	62.0	(57.3 to 66.4)	583, 414	62.7	(58.5 to 66.7)	745, 417	0.8170
Friends of about own age	68.5	(62.9 to 73.5)	381, 278	77.7	(73.1 to 81.7)	491, 268	0.0093
First girlfriend/boyfriend/sexual partner	75.0	(67.0 to 81.6)	185, 132	82.3	(73.5 to 88.7)	113, 62	0.1713
Mother/Father	78.0	(68.5 to 85.3)	105, 78	68.3	(61.7 to 74.2)	280, 154	0.0854
Media	70.7	(59.8 to 79.6)	99, 75	77.9	(68.6 to 85.0)	127, 79	0.5031
Internet (excluding pornography)	74.5	(61.5 to 84.3)	65, 47	81.1	(60.1 to 92.4)	37, 23	0.2612

*Denominators are presented as unweighted, weighted.

### Preferred source of additional information

**Figure 1:**
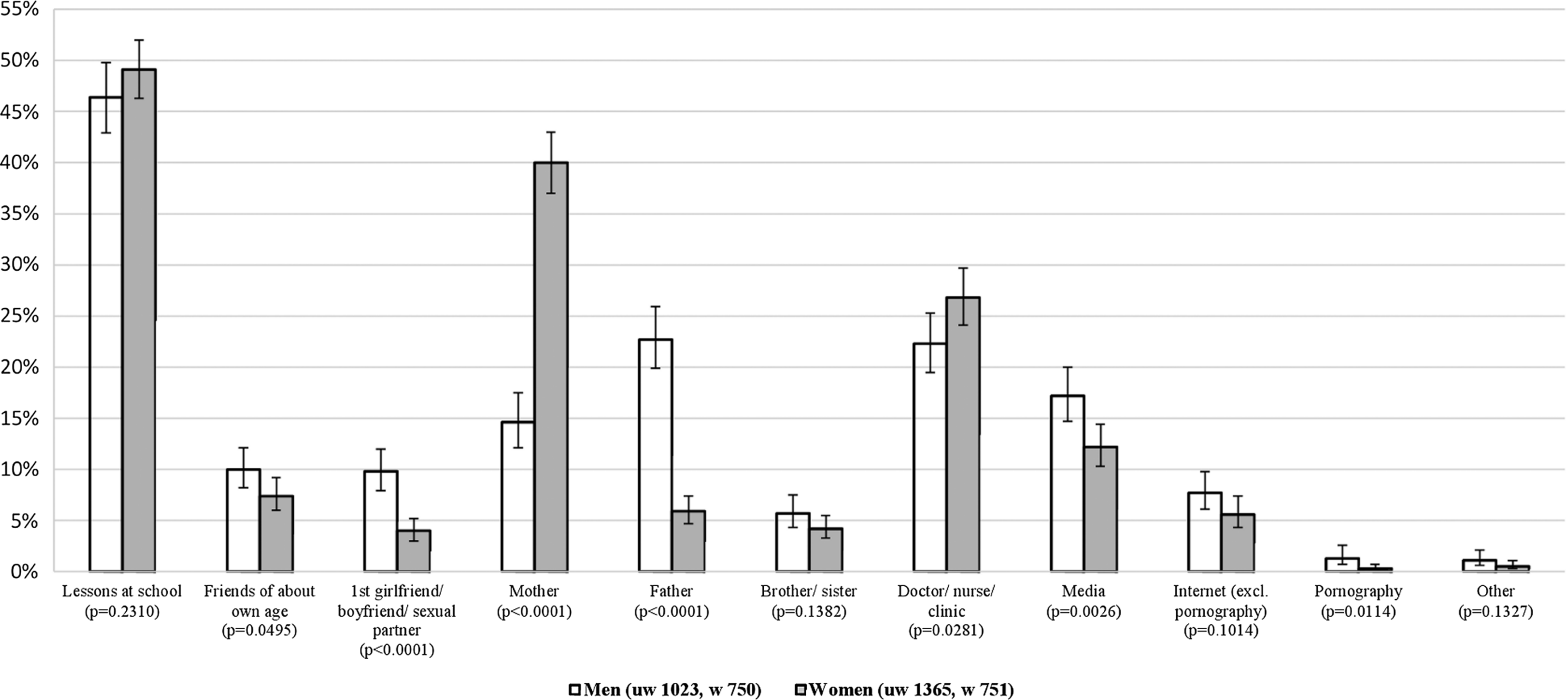
Preferred source of additional information about sex in individuals aged 16–24 years in Natsal-3 who felt they ought to have known more, by gender.

Among those who felt they ought to have known more, school was the most commonly-reported preferred source for both men and women (47.7%, no gender difference; figure 1), followed by mothers for women (40.0%) and fathers for men (22.7%). A sizable proportion of men also reported their mother (14.6%). Young people also commonly wanted information from health professionals (22.3% of men and 26.8% of women) and many mentioned media sources (17.2% of men and 12.2% of women). Other sources were reported by 10% or fewer participants.

### Range of sources of information about sex and unmet information needs in men reporting pornography as one of their information sources

The 23.9% of men for whom pornography was a source of information about sex reported a larger number of sources (a median of 5 vs 3 for men not reporting pornography as a source of information; p<0.0001) and were more likely to report their friends, first sexual partner, siblings, media and the internet (excluding pornography) than men who did not report pornography as a source of information (table 4). These men were less likely to report lessons at school as one of their information sources. Men citing pornography as a source were less likely to report lessons at school or their mother as their main source of information and were more likely to cite their first sexual partner. They were also more likely to have unmet information needs (76.2% (71.1 to 80.6) vs 65.5% (62.4 to 68.4)), more frequently reporting needing biological (28.6% (23.8 to 34.0) vs 20.1% (17.5 to 22.9)) and psychosexual information (53.4% (47.7 to 59.1) vs 37.7% (34.7 to 40.9)) than men for whom pornography was not an information source.

**Table 4:** Other and main sources of information about sex and adequacy of information in men aged 16–24** **years in Natsal-3 by whether they reported pornography as a source of information about sex or not

	Pornography mentioned as a source		Pornography not mentioned as a source		Logistic regression		
	Per cent	95% CI	Per cent	95% CI	OR*	95% CI	p Value
Other sources of information about sex							
Lessons at school	72.7	(67.9 to 77.0)	80.3	(77.6 to 82.7)	0.65	(0.49 to 0.87)	0.0033
Friends of about own age	75.3	(70.3 to 79.7)	63.0	(60.0 to 65.9)	1.79	(1.36 to 2.36)	<0.0001
First girlfriend/boyfriend/sexual partner	40.6	(35.2 to 46.3)	24.0	(21.5 to 26.7)	2.16	(1.65 to 2.83)	<0.0001
Parents	28.8	(24.4 to 33.7)	26.7	(24.0 to 29.5)	1.11	(0.85 to 1.46)	0.4350
Mother	19.5	(15.8 to 23.8)	19.9	(17.6 to 22.5)	0.97	(0.72 to 1.32)	0.8600
Father	20.0	(16.3 to 24.3)	16.8	(14.7 to 19.3)	1.24	(0.92 to 1.67)	0.1602
Brother/sister	14.0	(10.4 to 18.7)	9.6	(7.8 to 11.8)	1.53	(1.03 to 2.29)	0.0364
Doctor/nurse/clinic	8.2	(5.6 to 11.9)	6.5	(5.3 to 8.1)	1.27	(0.80 to 2.03)	0.3095
Media	65.5	(59.6 to 71.0)	41.2	(38.2 to 44.3)	2.71	(2.05 to 3.60)	<0.0001
Books/magazines/newspapers	31.2	(26.3 to 36.5)	16.7	(14.5 to 19.1)	2.27	(1.70 to 3.03)	<0.0001
TV/radio/DVDs/videos	57.7	(51.9 to 63.4)	33.7	(30.9 to 36.7)	2.68	(2.05 to 3.52)	<0.0001
Internet (excluding pornography)	47.6	(41.9 to 53.3)	22.7	(20.0 to 25.6)	3.09	(2.31 to 4.13)	<0.0001
Internet-sexual advice websites	32.0	(27.1 to 37.4)	15.0	(12.8 to 17.5)	2.66	(1.94 to 3.66)	<0.0001
Internet-other	26.7	(21.8 to 32.1)	10.1	(8.5 to 12.1)	3.22	(2.30 to 4.53)	<0.0001
Other	0.8	(0.3 to 2.2)	1.7	(1.1 to 2.6)	0.49	(0.17 to 1.41)	0.1868
Main source of information about sex							
Lessons at school	24.6	(19.9 to 30.1)	44.0	(41.0 to 47.0)	0.42	(0.31 to 0.56)	<0.0001
Friends of about own age	26.1	(21.8 to 31.0)	23.5	(20.9 to 26.2)	1.15	(0.87 to 1.53)	0.3147
First girlfriend/boyfriend/sexual partner	16.1	(12.3 to 20.8)	10.0	(8.3 to 12.0)	1.73	(1.20 to 2.51)	0.0035
Mother	1.8	(1.0 to 3.2)	5.1	(3.8 to 6.6)	0.34	(0.17 to 0.67)	0.0021
Father	4.0	(2.3 to 7.0)	2.5	(1.6 to 3.7)	1.66	(0.82 to 3.39)	0.1614
Brother/sister	1.9	(1.0 to 3.8)	2.2	(1.5 to 3.2)	0.88	(0.40 to 1.97)	0.7641
Doctor/nurse/clinic	0.7	(0.2 to 2.1)	1.0	(0.5 to 1.8)	0.65	(0.17 to 2.47)	0.5284
Media	5.5	(3.6 to 8.5)	6.9	(5.4 to 8.7)	0.79	(0.47 to 1.35)	0.3952
Internet (excluding pornography)	4.9	(3.0 to 7.9)	3.9	(2.9 to 5.1)	1.27	(0.71 to 2.29)	0.4187
Pornography	14.2	(10.8 to18.5)	0.0	–	NA	–	–
Other	0.1	(<0.1 to 1.0)	1.1	(0.6 to 2.0)	0.13	(0.02 to 0.99)	0.0486
Adequacy of information about sex							
Needed more information	76.2	(71.1 to 80.6)	65.5	(62.4 to 68.4)	1.69	(1.26 to 2.26)	0.0005
Needed biological information	28.6	(23.8 to 34.0)	20.1	(17.5 to 22.9)	1.60	(1.18 to 2.16)	0.0023
Needed risk-reduction information	48.0	(41.9 to 54.1)	45.4	(42.3 to 48.6)	1.11	(0.84 to 1.46)	0.4641
Needed psychosexual information	53.4	(47.7 to 59.1)	37.7	(34.7 to 40.9)	1.89	(1.45 to 2.47)	<0.0001
Unweighted, weighted denominators	400, 296		1328, 942				

*ORs compare odds of reporting each source/information need in those mentioning pornography to the odds of reporting each source/information need in those not mentioning pornography.

### Change over time

**Figure 2:**
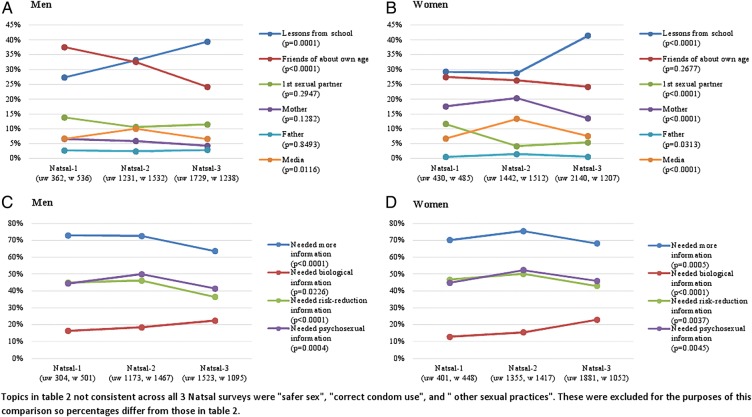
Change over time in main source of information about sex (A and B); and topics young people felt they ought to have known more about (C and D).

Since 1990–1991, there has been a large increase in the proportion of men and women citing school as their main source of information about sex (figure 2A), from around 30% in 1990–1991 to around 40% in 2010–2012. There has been a corresponding decrease in the proportion of young men reporting friends and in the proportion of young women reporting their first sexual partner as their main source of information. While there has been no change in the proportion of men reporting a parent, there has been a decrease in the proportion of women reporting their mother (but not their father) since 2000.

Between 1990–1991 and 2010–2012 there has only been a modest decrease in the proportion of men reporting needing more information about sex (figure 2B) and in women, no decrease. The proportion of men and women who reported needing biological information has increased but, since Natsal-2, undertaken in 1999–2001, there has been a decrease in the proportion reporting needing risk-reduction or psychosexual information.

## Discussion

### Highlights

These data show a marked increase over recent decades in the proportion of young people citing school as their main source of information about sex, with a corresponding decrease in the proportion reporting friends (men) and first sexual partners and mothers (women). Despite this, there has been no major change in perceived adequacy of information and young people continue to feel they need more risk-reduction and psychosexual information, although there has been some decline in the proportion who would have liked additional risk-reduction information. Predominantly, young people wanted this additional information from school, parents and health professionals. Parents and health professionals were relatively rarely cited as a main source of information in spite of being commonly reported as a preferred source, particularly for men.

### Strengths and weaknesses of the study

The major strength of this study is that it uses data from serially-conducted population-based surveys with nationally representative samples. We are able to examine change over time as these surveys employed the same methods and measures. The sample of young people is large, providing robust estimates from 3869 men and women in Natsal-3 and a total of 7334 young people from all three surveys in whom we have explored change over time. Using data from the three surveys to examine change over time (as opposed to looking at change across the life course in one survey) is likely to minimise recall and recasting biases. The response rate for the latest survey (Natsal-3) was 57.7%, which is comparable with other population-based surveys completed around the same time.^29^ ^30^ After weighting our data to match the British population for age, gender and geographic region, the sample was comparable with the 2011 census data on other key demographic characteristics.^27^ Given the rapid evolution of technology, one limitation is that some of the Natsal-3 participants included in this analysis would have been learning about sex around the beginning of the 21st century and their experience will be different from those learning about sex today with respect to internet access, particularly through smart phones. Another limitation is that we used a predefined list of topics to determine further information needs, which may not have reflected adequately the range of topics of interest to young people today. We also do not know about the quality of the information that young people received.

### Strengths and weaknesses with respect to other studies and important differences in results

A larger proportion of young people in our study reported school as their primary information source than reported school as one of their three main sources in an internet-based survey of young people in the UK.^13^ However, our findings are consistent with those from smaller studies of young people which report further information needs from school-based sex education^11^ ^13^ and observations such as those from Ofsted that too much focus was placed on ‘the mechanics’ of reproduction with too little teaching about relationships^16^ and that large proportions of gay/lesbian, bisexual young people report inadequate SRE.^12^ ^31^ ^32^

### Meaning of the study, possible explanations and implications for clinicians and policymakers

According to our data, pornography is a source of information about sex for nearly one in four men as they are growing up. Concerns from young people, particularly women, about the negative effect of pornography on the way that young people are expected to look and behave highlight the importance of the recent supplementary SRE advice^22^ to enable those teaching SRE to challenge images and norms portrayed through pornography.

Nevertheless, pornography is a *main* source for only a small proportion of young men and a very small proportion of women, and, despite the availability of the internet, young people still most frequently report more traditional sources of information about sex (school, friends and parents). The increase in reporting of school as the principal source of information about sex is encouraging as school-based sex education has the potential to reduce inequalities in the provision of information. Respondents reporting school as their main source were less likely to report wanting further information. In other analysis we found that reporting school as a main source of information about sex was associated with lower reporting of a broad range of sexual health outcomes, particularly among women.^33^ ^34^ However, despite this increase, there has been no substantial change in young people's information needs. This should be considered in the context of the changing social climate of young people's lives but our research suggests that a gap remains between what young people want to know about sex and what is covered by current requirements for SRE. Results of other studies and reports support this. This suggests that the quality and breadth of SRE in schools needs to be improved, particularly since young people in our survey most frequently reported wanting this additional information from school. Recent polls also suggest strong support for SRE among parents.^35^ ^36^

Having a parent as a main source of information is the experience of only a minority of young people. Yet second to school, young people reported parents as their preferred source of additional information and this finding echoes data from other research showing that many young people felt that responsibility for teaching about sex and relationships should be shared between parents and teachers.^13^ Furthermore, parental involvement in their children's sexual learning remains strongly gendered. The gap between the proportion of young people preferring to receive information from their parents and the proportion actually doing so is particularly large for men, and particularly from fathers. Some researchers suggest that fathers may leave talking about sex to mothers believing that they have better interpersonal skills.^37^ An open dialogue about sex between parents and children may be difficult for many reasons.^37–41^ Yet most parents feel that home and school should both have a role in SRE.^42^

The disparity between the proportion of young people wanting, and receiving, information about sex from health professionals also warrants attention. The school nursing service may play an important role in SRE.^43^ Government SRE guidelines^2^ also highlight the potential for health professionals to support SRE teaching and make links between schools and other health services (eg, family planning or sexual health services). The move, in England, to placing responsibility for public health, including the commissioning of sexual health services, within local authorities^44^ ^45^ may provide the opportunity for closer coordination between PSHE and sexual health services.^46^

### Unanswered questions and future research

Further research is needed to understand how communication about sexuality between parents/carers and children can be encouraged and supported, particularly for sons and fathers, and taking into account changes in family structure. Further research is also needed to understand how young people would like to receive information about sex from health professionals, for example, in a one-to-one setting or through school SRE. Many unanswered questions remain around the influence of pornography on young people.^20^

It is unclear what impact the increase in academy and free schools, which do not have to follow the National Curriculum, will have on the provision of school-based SRE and whether gains made will be lost. The Healthy Schools Programme, which placed importance on good PSHE in schools, changed in 2010 with responsibility for implementation and monitoring now at school or locality level, with no requirement or national support for local implementation. This may lead to less focus on PSHE within schools. Finally, the outcome of the Education Select Committee inquiry on PSHE and SRE^47^ may impact on the role of schools in teaching about sex.

### Conclusion

School lessons have, over the past 20 years, become the main source of information about sexual matters for increasing proportions of young people in Britain. Nevertheless, the majority continue to report needing more information on a broad range of topics, most of which are not covered by current requirements for SRE. Preferred sources of this information are school, parents and health professionals, despite the latter two being less frequently reported as information sources, particularly by men. These data support a broadening of the statutory requirements for SRE provision and suggest the need for additional support for parents in helping them to take an active role in teaching their children about sex and relationships.
